# The complexities of postcolonial international health: Karl Evang in India 1953

**DOI:** 10.1017/mdh.2023.12

**Published:** 2023-01

**Authors:** Sunniva Engh

**Affiliations:** Department of Archaeology, Conservation and History (IAKH), University of Oslo, Oslo, Norway

**Keywords:** Karl Evang, World Health Organization, Postcolonial health, India, Social medicine, International health

## Abstract

In February and March 1953, a WHO Visiting Team of Medical Scientists worked in India, collaborating with local medical students and professionals. This article studies the complexities of early postcolonial international health work and the relations between the young WHO and the newly independent countries, from the position of the team’s vice chairman, Norwegian doctor Karl Evang. While the WHO aimed to create dialogue and interaction, also learning from the host country, the article finds that an equal exchange of views between visitors and hosts was not achieved. The topic pertains to discussions on power and influence in international organisations and governance, development and health work, within a South Asian setting. Studying intellectual exchanges between Evang and his Indian interlocutors sheds light on India’s role as both receptive and generative site of ideas and political practice, contributing to broader debates on the appropriation, refashioning and application of political ideas in independent India. Also, at a time of new directions in international health, and considering Evang’s social medicine conviction, an additional question concerns the role of social medicine. The article underlines the existence of multiple, parallel tracks in international health work, and argues the need to portray international health as a complex mosaic, rather than a step-by-step development. The case has relevance as historians endeavour to make international and global history more diverse, as through Evang we capture parts of a broader international involvement of people and nation states in the WHO and its work in the early post-war period.

…the month of February 1953…was the month when the world was with us. …For four hectic weeks the college rang with voices from far corners of the globe and an intellectual camaraderie reigned in our halls. We refer to the visit of the W.H.O. team of medical scientists …. Lectures, demonstrations, operations, discussions – a delectable feast for the mind.… New vistas were opened up to us and before us lay the dazzling treasures of the New World.…

But we are sure the East renowned for its wisdom, did shed some light on the path of learning that our visitors pursue.…Many tropical diseases that were but words in textbooks for them acquired a meaning. Labouring under the lack of facilities, battling against heavier odds and with the low living standards that impede progress, we yet held our own.^1^

In February and March 1953, a World Health Organization (WHO) team of high-profile medical scientists visited India, interacting with medical students, professionals, institutions and politicians in New Delhi, Madras and Bombay. Having grown out of a series of ‘Medical Teaching Missions’ originally organised by the United Nations Relief and Rehabilitation Administration’s (UNRRA) and carried out under the aegis of the WHO Interim Commission from 1947, the Visiting Team of Medical Scientists became the first of its kind. The teaching missions’ core idea had been to re-integrate the medical establishments of previously German-occupied countries with Western medicine, following World War II. Reflected in the change of name, WHO medical visitors were no longer supposed to be on a ‘mission’ to disseminate Western knowledge. On the contrary, the aim was an exchange of views, ideas and research findings, while also learning from the host country. As the WHO pointed out to the Team members, ‘…such visits really amount to an effective exchange of scientific information instead of being one-sided teaching.’^2^ Indeed *The Madras Medical College Magazine*’s editorial on the Visiting Team, quoted above, leaves impressions of the Team’s inspirational medical work, whilst also underlining the Indian contribution to the team members’ learning.

From the vantage point of Karl Evang (1902–1981), the WHO Visiting Team’s vice chairman, we may gain insights into the complexities of and influences on early post-war international health work. A Norwegian medical doctor, radical and disseminator of social medicine, Evang played a key role in international health work in the immediate post-war period, as one of the instigators of the WHO,^3^ an organisation to which he was particularly committed.^4^ As Norway’s Director of Health (1938–1972), Evang’s impact on Norway’s national health administration was also momentous.^5^

Using a biographical approach, this article explores what Evang’s work in and impressions from India may reveal about cooperation between the young WHO and the newly independent countries in the early 1950s. The time brimming with ideas and initiatives in health and development, such cooperation nevertheless took place in contested waters; the WHO visit happened only six years after India’s independence, but it was still ahead of the main wave of decolonisation. We query, as gleaned through Evang’s work, did the WHO’s Visiting Team represent an actual change from the earlier Teaching Missions, creating dialogue, interaction and an equal exchange of views, or was the change rather a semantic one? Also, in a period of changing directions and discourses in international health, what was the place of social medicine?

This article pertains to ongoing discussions on power and influence in international organisations and governance, development and health work,^6^ particularly within a South Asian and Indian setting.^7^ South and Southeast Asia were not only central, challenging sites for international interventions in health and development, but according to Sunil Amrith, also the most ardent supporters of such, particularly those of the WHO.^8^ At the same time, in such interventions, South Asian politicians, medical professionals, voluntary agencies and entire communities played considerable roles, negotiating and limiting the reach of organisations such as the WHO.^9^ Thus, the topic resonates with broader debates on the international transfer of ideas and the importance or not of the appropriation, refashioning and practical application of political ideas in independent India.^10^ As such, looking at the intellectual exchange between Karl Evang and his Indian interlocutors, the article may shed light on India’s role as both receptive and generative site of ideas and political practice^11^ in health and development, in its encounter with the WHO Visiting Team of Medical Scientists.^12^

Considering Evang’s strong commitment to social medicine, an additional main question revolves around the role and legacy of social medicine in early post-war international health cooperation. According to Iris Borowy, social medicine ‘reached its apex’ between the mid-1930s, the heyday of the League of Nations Health Organization (LNHO), and 1948, the WHO’s first year.^13^ Sunil Amrith has pointed out that, despite its preamble reflecting the organization’s social medicine heritage, visions of social medicine were soon given less room in the WHO, as a narrow, technology-focused approach grew forth in the early post-war years.^14^ Similarly, Marcos Cueto, Theodore M. Brown and Elizabeth Fee’s recent history of the WHO argues that social medicine was practically put aside as the organisation, following the Soviet Union’s 1949 withdrawal, rapidly embraced ‘technical assistance,’ also becoming increasingly influenced by US interests.^15^ Thus research on international health and the WHO may implicitly assume a step-by-step, incremental development of dominant approaches in international health, where one approach seemingly yielded to the next as its influence was waning, and other, newer fashions appeared. In contrast, this article argues the need to acknowledge the multiple, parallel tracks in international health work, complicating the narrative and portraying international health work as a complex mosaic, rather than a step-by-step development. Thus, social medicine did not simply disappear from the international health agenda as vertical eradication programmes became fashionable – indeed, some milieus, such as Evang’s, still saw it as the cutting edge of medicine – whilst vice versa, when social medicine ‘reappeared’ with the 1978 Alma-Ata declaration, vertical eradication programmes still had considerable impact.

Thus, a look at early WHO work from Evang’s vantage point may nuance our understanding of social medicine in the organisation’s early years. A use of Evang as a lens on this period seems warranted for three reasons. Firstly, Evang was one of a handful key actors in the early WHO. Secondly, he was a long-time propagator of social medicine, both at home and abroad – and in Evang’s Norway, social medicine was on the increase rather than in decline during the post-war years, as the social democratic welfare system was gradually consolidated. Thirdly, his experiences with the 1953 WHO Visiting Team to India show how Evang actively worked to promote social and preventive medicine, attempting to counter contemporaneous currents in international and Indian medical work.

Arguably, the case also has relevance as historians endeavour to make international and global history more diverse, seeking to include the voices of historical actors originating outside Western large powers and beyond the anglophone world, aiming to rectify earlier biases.^16^ Such inclusion may fruitfully complement, nuance and ultimately also challenge the dominant narratives of development, health and international relations. Moreover, whilst key roles often were held by great powers and previous colonisers, even Norway, a small country on the margins of Europe, with limited colonial experience, took part. For example, in 1952, Norway, as the second country in the world, launched a bilateral aid project: a fisheries and health aid project in Kerala, India – where Evang also held a central role.^17^ As Patricia Clavin has pointed out, international engagement could, for small countries, strengthen agency and define identity, and their inclusion may help decentre international history, off-setting a bias towards larger countries.^18^ Thus, through Evang we capture parts of a broader international involvement of people and nation states in the WHO and its work in the early post-war period.

The following article provides an overview of Evang’s background and entry into international health, before turning to the 1953 WHO Visiting Team of Medical Scientists to India, and more particularly, Evang’s ambitions to strengthen social and preventive medicine in India’s health sector and medical education. Whilst located in the international literature, the article draws extensively on Norwegian sources and archives, aiming to contribute to the widening of international health history’s scope,^19^ but at the same time acknowledging its relative lack of Indian sources on the WHO’s Visiting Team of Medical Scientist.

## Karl Evang: radical early years

Throughout his life, Karl Evang’s work was marked by a few, core influences: a commitment to radical leftist politics, a firm grounding in social medicine and a commitment to public health. Enrolling in medical studies in Oslo in 1924, Evang soon joined the radical socialist association *Mot Dag* ^20^ and the Clarté pacifist movement. Whilst serving a prison sentence in 1931 for refusing national military service, Evang published a book on birth control.^21^ Allied with radical feminist movements, Evang saw access to contraception and abortion as crucial for improving women’s health.^22^

Evang’s social medicine interest was spurred by studying Alfred Grotjahn’s *Soziale Pathologie* [Social Pathology].^23^ In the inter-war years, social hygiene and social medicine were influential directions in Norwegian medicine, reflected in an increase in published research on the social aspects of health.^24^ Several Norwegian physicians trained in German universities and read German academic literature, just as ‘the founder of social medicine,’ Rudolf Virchow (1821–1902) and Alfred Grotjahn (1869–1931) were influential. Carl Schiøtz (1877–1938), professor of hygiene and bacteriology at the University of Oslo from 1931, was a particular promoter of social hygiene and social medicine,^25^ with connections through the League of Nations, Germany and the USA.^26^

In 1931, Evang attended a conference on public health organised by the German *Verein Sozialistischer Ärtze.* [The Association for Socialist Physicians],^27^ and he promptly founded a Norwegian branch, *Socialistiske Lægers Forening* [Association for Socialist Physicians], which was followed by Swedish and Danish branches in 1933.^28^ Under Evang’s leadership, the association became a key promoter of social medicine in Norway.^29^ Grotjahn’s core idea that ‘between man and nature is culture’ became a life-long dictum of Evang, who argued that societal changes were necessary to optimise health and minimise illness. To Evang, medical science was a *social* science, breaking with medicine’s focus on the individual. Evang’s approach to social medicine entailed both a *politicisation* of medical science, and a *scientification* of politics.^30^ *Sosialistiske Læger* was a medical and political project, aiming to ‘socialise’ the medical profession whilst increasing its political influence. The socialist society was the ideal, with a ‘complete societal take-over of businesses that secure the population’s health’.^31^

Evang’s commitment to sexual health led to the 1932 founding of *Populært Tidsskrift for Seksuell Oplysning* [Popular Journal for Sexual Enlightenment],^32^ which soon spurred Swedish and Danish editions. Birth control campaigner Elise Ottesen-Jensen was central in the Swedish edition, and in cooperation with Swedish socialist physicians, she established the *Riksförbundet för Sexuell Upplysning* (RFSU) [The Swedish Association for Sexuality Education] in 1933.^33^

During 1933–35, Evang conducted social medicine-inspired research on diet and living conditions in poorer households,^34^ which eventually brought Evang to the 1937 and 1938 League of Nations meetings of representatives of National Nutritional Councils.^35^ The League’s nutrition work being influenced by social medicine, ^36^ this involvement must be expected to have suited Evang’s interest, possibly even showing social medicine’s relevance on an international scale. Indeed, social medicine was fast becoming a key direction in international health work, as shown by several studies of the LNHO and other actors in inter-war health, with the 1937 LNHO conference in Bandung, Indonesia, marking a key event.^37^

In the late 1930s, Evang took on a central role in domestic government. Evang had joined the Norwegian Labour Party, which, during the mid-1930s, evolved from a revolutionary-inspired opposition movement into a broad mass movement. Gaining political power in 1935, Labour retained political dominance until the mid-1960s. As Labour shed its revolutionary past, Evang’s social medicine ideas also evolved: he no longer saw the state as a potential system of repression, but on the contrary, as a core enabler of health, a system to secure and guarantee the population’s health. Appointed Director of Health in 1938, the top administrative position in the Norwegian health system, Evang was given political and administrative powers to put his ideas into action.^38^ Evang’s appointment thus secured support for social medicine at the very top of the Norwegian health system, making the decades following World War II a period of growth for social medicine in Norway.^39^ It was on Evang’s watch that social medicine became a distinct, prioritised field of medicine at the University of Oslo, with the establishment of a new professorship and a Department of Social Medicine in 1951.^40^ Before these developments could take place, however, World War II would greatly increase Evang’s involvement in international health efforts.

## International health politics: Karl Evang’s role

When Germany occupied Norway in 1940, Evang escaped to London with the Norwegian government. From London, he established a Directorate of Health supporting Norwegians abroad. The London years and his frequent visits to Washington increased Evang’s international medical network and his enthusiasm for British social legislation and American public health developments.^41^ Also, while previous Norwegian Directors of Health had limited international health collaborations, eg. with the Rockefeller Foundation since the mid-1920s,^42^ the war time exile placed Evang squarely at several arenas where the post war international order was being shaped and where he threw himself into international health work.

In 1943, Evang attended the UN Conference on Food and Agriculture in Hot Springs, and he was Norway’s representative to the second session of the United Nations Relief and Rehabilitation Administration’s (UNRRA) Council in Montreal in 1944.^43^ Through UNRRA, Evang engaged in discussions of the future organisation of international health. At an October 1944 meeting of the American Public Health Association, Evang – together with UNRRA’s Deputy Director of Health James Crabtree and colleagues Szeming Sze and Geraldo de Paula Souza – debated ‘Today’s Global Frontiers in Public Health.’ The war had created setbacks in health work, and discussion centred on how best to organise future international health efforts. According to Evang, pre-war international health work had been fruitful, but he cautioned against a multiplicity of organisations: ‘in the post-war period we should endeavour to unify and centralize.’ Talk was underway of a new international health organisation, and Evang argued: ‘We have, in my opinion, to plan the coming international organization in such a way that there will be no interval, no lapse, between the termination of UNRRA’s work and the beginning of the work of the other. To meet this situation, we have to plan now.’^44^

As part of the Norwegian delegation to the 1945 San Francisco conference,^45^ Evang had the opportunity to do precisely that. It was there, at the United Nations Conference on International Organization, that Evang, Sze and de Paula Souza planned a joint initiative, which eventually paved the way for the World Health Organization.^46^ Evang became central in the preparatory work for the WHO, as one of the 16 members of the Technical Preparatory Committee for the International Health Conference. Evang chaired Norway’s delegation to the International Health Conference in New York in 1946 and was a member of the WHO Interim Committee 1946–1948. From the first World Health Assembly in Geneva (1948) onwards, Evang chaired all Norwegian delegations to the yearly assemblies until his retirement in 1972, and he was President and Vice-President at the second and fourth World Health Assemblies in 1949 and 1951.^47^ Evang was, according to himself, several times strongly encouraged to take on central WHO positions, such as one of the assistant Director General positions, but he prioritised his duties as Norway’s Director of Health.^48^ Indeed according to Sze, Evang was considered as a potential first WHO Director General but was quickly ruled out as too controversial due to his support for birth control.^49^

As shown above, Evang’s social medical convictions had, from the early 1930s, led him to work within both nutrition and birth control. Now, in the early post-war years, Evang had a hand in the UN’s nutrition work through the FAO’s Standing Committee on Nutrition^50^ and its health work through the WHO. The involvement in the new UN system of organisations thus gave Evang opportunity to work with these interrelated issues on a global level. Evang drew on the works of William Vogt, Fairfield Osborn and Georg Borgström, among others.^51^ As an early supporter of extending family planning advice to ‘Third World’ countries, his views coincided with those of a growing number of supporters of neo-Malthusianism.^52^ The core concern was that the populations of the developing countries was increasing rapidly, and should it remain unchecked, the assumed result would be resource shortages, famine and conflicts, potentially jeopardising the newly won world peace.

At the 1952 World Health Assembly, Evang raised the population issue, arguing that the WHO should establish an expert committee ‘on the health aspects of the population problem’ and take part in the 1954 World Population Conference. While health assemblies in 1948 and 1950 had agreed that collaboration with the UN on population matters should be continued and developed, Evang argued it was time to take a further step.^53^ While the proposal met with vast opposition from several Catholic countries and hesitation from a number of Western countries, Evang had the support of core WHO staff including Brock Chisholm, as well as the delegations of Sweden and India, amongst others.^54^

Evang’s willingness to raise the population issue secured him notoriety in some circles, at the same time cementing other relations. Sweden’s representative at the 1952 Health Assembly, Axel Höjer (1890–1974), was Evang’s opposite number as Sweden’s Director of Health (1935–1952) and a close friend. In autumn 1952, Höjer and his wife Signe moved to India to take up a WHO-funded post as Principal of the Medical College at the University of Travancore. The Höjers became important points of contact for Evang on India’s medical sector. Likewise, India’s head of delegation at the 1952 Assembly, Sir Arcot L. Mudaliar, also became a key connection. Indeed, Evang, Höjer and Mudaliar were also colleagues on the WHO Executive Board, where they all served in the early 1950s; Evang in the first, second and third sessions; Mudaliar in the third, fourth and fifth; and Höjer in the fourth and fifth sessions.^55^ In addition, Höjer and Evang shared a background in the League of Nations nutrition work and were also on the FAO’s Standing Committee on Nutrition together,^56^ thus illustrating how Scandinavians working with the League often continued within the UN system, further demonstrating the ‘tendency of continuity of Scandinavian internationalism across the Second World War,’ as pointed out by Gram-Skjoldager, Ikonomou and Kahlert.^57^

## The 1953 WHO Visiting Team of Medical Scientists: background and purpose

In March 1952, Erwin Kohn, head of the WHO’s Section of Exchange of Scientific Information, requested Evang’s participation in a 1953 WHO ‘Medical Teaching Mission,’ planned for India or Indonesia. A recent three-month mission to Burma and Ceylon had, according to Kohn, been ‘very successful,’ and these ‘medical educational projects’ were expected to become ‘more or less of a standard feature in many of the Regions.’^58^ The mission would include a public health group, like one Evang had headed in a 1951 mission to Israel. Evang confirmed his interest, saying ‘it is certainly difficult under the present circumstances to point to any other task which would have higher priority.’^59^ Evang saw the WHO Medical Teaching Missions as a ‘direct, effective and not too expensive way of promoting positive help,’ underscoring that ‘It is becoming more and more obvious that we must intensify the constructive work in the underdeveloped areas.’^60^ By early April 1952, the WHO had settled that the Medical Teaching Mission would focus on India, mainly the Bombay and Madras areas.^61^

To prepare to share information with the mission team, various WHO offices and Government of India agencies, the WHO’s Section of Exchange of Scientific Information published a series of ‘Information Bulletins.’^62^ These were circulated to the team members, relevant WHO units, the WHO Regional Office in New Delhi and Indian government agencies. Medical Teaching Missions had originated after WWII, sponsored by the UNRRA and the Unitarian Service Committee (USC), a non-profit organisation, as an initiative to ‘re-establish contact between the scientists of the German occupied countries and those of the western world.’^63^ In 1946, Medical Teaching Missions had visited Poland and Czechoslovakia. With UNRRA activities ceasing in 1947, the WHO Interim Commission had organised missions to Austria, Poland, Finland and the Philippines. In 1951, Medical Teaching Missions visited Israel and Iran.

The 1953 the WHO mission to India built on these previous missions, but with one important change; the change of name to ‘Visiting Teams of Medical Scientists.’ The first WHO Information Bulletin, distributed in May 1952, underlined that the purpose was ‘to bring leading scientists of various countries into personal contact’ so that they could exchange ideas, and ‘stimulate scientific endeavours both in the host and the home countries of the visiting scientists.’ Thus, the visits were intended as ‘effective exchange of scientific information’ rather than ‘one-sided teaching.’ Indeed, according to WHO’s Information Bulletin No. 1, previous Teaching Missions had shown that team members ‘learn a great deal during their visits to foreign countries and benefit greatly from the more thorough knowledge of their local colleagues of conditions and diseases peculiar to their countries or areas.’^64^

The 1953 Visiting Team was planned to comprise a ‘teaching staff’ of 15, plus administrative staff. There would be

three representatives of the basic medical sciences (physiology, pharmacology and microbiology), eight representatives of various clinical specialities (internal medicine, paediatrics, general surgery, orthopaedic surgery, thoracic surgery, obo-rhino-laryngology, anaesthesiology and radiology), a public health group of three (public health administration, epidemiology and sanitary engineering) and a specialist in medical education.^65^

The team included several big names in medical research, such as Sir Alexander Fleming, Professor of Pharmacology Corneille Heymans (both Nobel laureates), the orthopaedic surgeon Henry Osmond-Clarke, Professor of Physiology Einar Lundesgaard and Professor of Public Health Edward Grzegorzewski.^66^ Professor of Paediatrics Samuel Levine chaired the team, with Karl Evang as vice chair in charge of the public health group. In total, nine of these medical researchers had participated in previous WHO Teaching Missions. The team was the joint responsibility of the WHO Regional Office for South-East Asia, its Director C. Mani, and the WHO Headquarters at Geneva, with its Division of Education and Training Services directed by E. Grzegorzewski and the Section of Exchange of Scientific Information led by Erwin Kohn.

In Information Bulletin No. 2, issued in June 1952, the WHO again underlined the name change. By changing from ‘Medical Teaching Mission’ to ‘Visiting Team of Medical Scientists,’ the WHO wanted to eliminate two words they found misleading: ‘teaching’ and ‘mission.’^67^ The WHO wished to avoid any misunderstandings that the projects entailed a one-sided teaching effort, and ‘the erroneous impression as though these projects had some missionary character. Nothing could be farther removed from their real purpose.’^68^ Evang underlined the Bulletin’s statement that part of the team’s most important work was to demonstrate how ‘pre-clinical, clinical, and public health specialists work together in pursuing common aims’ and ‘how the preventive approach of the public health specialists has to find its legitimate place in any and every branch of the basic and clinical sciences.’^69^ To Evang, as chair of the public health group, the appeal of such integration of public health was clear; however, these statements may also indicate a continued focus on demonstration and teaching in the WHO’s approach.

The same Bulletin set out the Visiting Team’s programme in India and featured a text on medical education in India, by Sir Arcot L. Mudaliar, who, as Vice-Chancellor of the University of Madras, was a key member in the Madras Preparatory Committee. Throughout 1952 and 1953, further Information Bulletins provided background on India’s history, society and culture, as well as the Madras and Bombay preparatory committees and local medical professionals.^70^

## The role of public health: disagreements between the Government of India and the WHO Visiting Team

In late 1952, the Indian side presented objections to the WHO plans for the role and composition of the Visiting Team, causing great consternation both in the WHO and among team members. The Government of India was not persuaded by the WHO’s focus on public health, was hesitant about the public health group altogether, and had flat out refused the inclusion of a sanitary engineer in the Visiting Team. To Evang, this seemed a like setback before the team had got off the ground. Rolf Eliassen – Professor of Sanitary Engineering at MIT and the team’s designated sanitary engineer – wrote to Kohn and Evang to express his dismay. Disappointed to have ‘learned of their (India’s) desire to have only medical men on this Team,’ Eliassen argued it was ‘difficult to understand their attitude.’ The Indian reluctance made Eliassen conclude that ‘Evidently they are not thinking as strongly in terms of public health as they are from the standpoint of clinical medicine.’^71^ Evang also reacted strongly, fearing that the team’s public health contribution would be ‘critically reduced,’ urging Kohn to ‘do everything within your power to have the Indian government change its attitude.’^72^ Evang had discussed the team composition with Sir Arcot L. Mudaliar at the 1952 Health Assembly, thinking the matter settled. John Gordon of Harvard’s School of Public Health, the team’s epidemiologist, joined the discussion, labelling the Indian decision ‘short-sighted,’ and telling Evang that they would have to work hard ‘demonstrating the place of prevention,’ as India was ‘not particularly enthusiastic’ about the public health team.^73^ An exasperated Kohn told Evang that ‘you do not know to what lengths we have gone in persuading the Indian Government to reconsider,’ but that in spite of ‘insistence and persuasion,’ the Indian side could simply not be swayed.^74^

Accepting defeat, Evang couldn’t help arguing that the decision was ‘a great pity,’ which would ‘change the character of the public health group and its work considerably,’ but at the same time adding ‘We are, of course, completely at the service of the Indian authorities and will be happy to try to adjust our activities to their wishes.’^75^ According to several WHO employees, the Government of India, experiencing an influx of international advisors, aid donors and development projects, was eager to avoid duplication of efforts, seemingly somewhat reluctant regarding public health efforts.^76^

Indeed, considering contemporaneous Indian health efforts, the Indian hesitation should perhaps not be seen as a neglect of public health. The report of the Bhore Committee, published in 1946, became the guiding framework of India’s health care following Independence. Providing overviews of the Indian health services, the report recommended improvements and emphasised social medicine as a ‘modern trend in the organization of health services,’ discussing its potential application in India.^77^ According to Amrith, the influence of social medicine on the Bhore Committee’s work was quite direct, in the form of a group of international consultants, including noted propagators of social medicine such as the University of Oxford’s first professor of social medicine John Ryle, Henry E. Sigerist of the Johns Hopkins School of Public Health, and John Grant of the Rockefeller Foundation.^78^ The Bhore report, as a result, appeared as a ‘clear endorsement’ of social medicine and emphasised that health care was a state responsibility and medical personnel should be public employees, ideas that resonated with social medicine.^79^ Indeed sources of inspiration for the Bhore Committee report, such as the 1942 Beveridge report,^80^ published whilst Evang worked in London, were also inspirational in Evang’s own work, influencing his scheme for Norway’s medical services following the war.^81^ The same also went for American plans for post-war health services, which Evang knew well through his stays at Johns Hopkins and Harvard Schools of Public Health, also during the war. ^82^ In 1944 Evang had argued that ‘In the United States and Great Britain extensive plans for medical organization and medical security are under discussion,’^83^ pointing these out as examples of positive developments in health. Berg identifies parallels between core ideas of the Bhore Committee report and those of Scandinavian social medicine, as set out in Axel Höjer’s publications, such as the key role of preventive health, an emphasis on nutrition and mother and child health, and the state’s responsibility for the citizens’ health.^84^

Considering the influx of foreign experts and consultants, the Indian government’s hesitation over the WHO Visiting Team may, rather, have reflected a wish to set the terms for the Team’s visit. Indeed, as Nikhil Menon has argued, while India received numerous experts from abroad, ‘several of the most influential individuals and institutions were home-grown,’ and Indian government officials and politicians had, of course, their own visions for modernisation and development, several of which grew out of colonial experiences.^85^ Jawaharlal Nehru’s government set India on a rather broad and ambitious path towards modernisation and development, which was to ‘make India economically independent via industrialization, solve India’s rural problems, especially the harsh inequalities and poverty, and provide the foundation for ‘democratic socialism.’^86^ Indeed, according to Zachariah, Indian visions for modernisation had a certain plasticity,^87^ accommodating both *dirigiste*, state-led approaches to development, for example through large-scale mechanisation efforts, whilst at the same time also encompassing broader grass roots or village-based development efforts, such as the 1952 ‘Community Development Programme.’^88^ As shown by Bhattacharya, the gap between plans, available resources and realities on the ground were considerable in Indian health policy in the years following independence, with disagreements at several administrative levels. Indian contributions to the WHO also went hand in hand with Indian ambitions to play prominent and independent international roles, whilst repeated requests for WHO assistance were a necessity.^89^

Tensions between the Government of India and the WHO were perhaps inevitable, considering the WHO’s ambitions for its Visiting Team and Indian preferences and wishes as a host country, which, as a recently independent nation, also navigated colonial legacies and influences whilst pursuing its own ideas on development. The WHO staff and Visiting Team members’ ‘insistence and persuasion’ and their frustration at the Indian government’s decisions thus indicate that visitors and hosts were not yet on an entirely equal footing, despite the WHO’s intentions to move away from earlier connotations of one-sided teaching missions.

## The Visiting Team’s work in India, and Evang’s focus areas

Arriving in India 5 February 1953, the WHO Visiting Team of Medical Scientists spent two months in the Bombay and Madras areas. After initial meetings and teaming up with Indian ‘counterparts’, a comprehensive programme followed. Most days consisted of a combination of lectures and seminars and work in local departments of medicine, followed by a detailed evening programme with film screenings, dinners, talks and social events.^90^ The Team also visited Poona and Delhi. While the Indian government had made the WHO reduce the Team’s public health group’s numbers from three to two, this made Evang all the more convinced of the need for public health work. His schedule prioritised public health and preventive medicine, including visits and collaborations with Indian counterparts.

In Delhi, Evang connected with top officials of public health, both at the state and union level and a whole host of others. Evang’s diary entries for his five days in the capital reveal a hectic schedule, comprising meetings with Rajkumari Amrit Kaur, Minister of Health of the Union, and K.C.K.E. Raja, Director-General of Health services, and his staff, as well as a press conference and meetings with Sushila Nayar, Minister of Health of the State of Delhi, Dr. Chadha, the Director of Health of the State of Delhi, and health officials of Delhi’s various municipalities.^91^ In addition, he participated in a dinner hosted by Amrit Kaur, five field visits to smaller health clinics, one speech, a round table discussion, a broadcast, meetings with India’s Planning Commission, the Ministry of Food and Agriculture and others, all topped with a reception and dinner at Rashtrapati Bhawan, the official residence of India’s President.

Public health and preventive health were overarching topics for Evang’s discussions with central Government of India representatives in Delhi, exemplified through his meetings with K.C.K.E. Raja. A central public health professional, Raja had been Secretary to the Bhore Committee,^92^ worked on the 1951 census and represented India on the WHO Regional Committee for South-East Asia. Evang received copies of the Bhore Committee’s report and Raja’s own text on ‘Teaching of Preventive Medicine’ in India, as he was currently writing a report on medical education^93^ – this would later form the basis for the All-India Institute of Medical Sciences in Delhi.

In contrast to the days in the capital, filled with high-level meetings, the Visiting Team’s stays in Madras and Bombay provided more time for field work. In Bombay, Evang worked daily alongside Dr V.V. Puri, Executive Health Officer of the Municipal Corporation of Greater Bombay, including visits to refugee camps, clinics for venereal disease and a number of laboratories, local clinics and medical colleges, taking part in discussions and seminars. In Bombay, Evang gave lectures on ‘The Scope of Public Health,’ ‘The Role of the Medical Practitioner in Carrying out Public Health Measures’ and ‘Solving a Community Health Problem;’ participated in a seminar on Medical Education and Social Medicine and discussions of health administration; and a broadcast on ‘Social Aspects of Medical Science.’^94^

Evang’s stay in Bombay also provided opportunity to familiarise himself with family planning work being done in the state, with visits to public, private and voluntary family planning clinics,^95^ which he found ‘exceedingly encouraging.’^96^ This part of Evang’s work had partly been prepared by the Höjers, after their move to India. According to Axel Höjer, Catholic influence made the Travancore-Cochin state government ‘not want to hear a word about birth control,’^97^ noting that, at central level ‘is our Gandhi-friend, the princess, who will absolutely not hear talk of birth control methods other than abstinence.’ Finding Minister of Health Rajkumari Amrit Kaur’s approach to the population matter insufficient, the Family Planning Association of India’s (FPAI) work seemed impressive by contrast, with 23 clinics in the Bombay area. The Höjers could also report that the upcoming Third International Conference on Planned Parenthood in November 1952, organised in Bombay by FPAI founder Dhanvanti Rama Rau, Margaret Sanger and C.P. Blacker, would also include Swedish birth control activists Elise Ottesen-Jensen and Niels Nielsen.^98^ Evang was impressed at how far the family planning work had come and agreed to be a sponsor of the Third International Conference on Planned Parenthood.^99^

Whilst missing the conference, Evang connected with the FPAI in March 1953, interacting with central members Rama Rau, Dr A.P. Pillay, Dr Hannah Peters and Dr Sushila Gore.^100^ At the Godfrey Clinic, Evang discussed birth control with Dr Gore, and at FPAI’s own clinic, Evang was shown around by Rama Rau, Dr Pillay and Dr Peters.^101^ At Rama Rau’s request, Evang lectured on ‘Health Problems in Relation to Human Reproduction’ at an FPAI meeting.^102^ Some of these activities being additional to his WHO programme, Evang clearly prioritised the interaction with the FPAI.^103^ Few archival traces remain of Evang’s discussions with FPAI members; however, Rama Rau’s views of population growth as a potential hindrance for economic development, and her emphasis on women’s right to regulate their fertility,^104^ should be expected to have resonated with Evang. In addition, connections between FPAI members and Evang’s colleague from *Populært Tidsskrift* Elise Ottesen-Jensen were very close in birth control activism, but they were also personal; Avabai Wadia, Hannah Peters and, above all, Sushila Gore, developed life-long friendships with Ottesen-Jensen.^105^

Evang’s campaigning of the population issue at the May 1952 World Health Assembly was noted by the FPAI and may have been behind the request for his sponsorship of the Third Planned Parenthood Conference.^106^ Writing to Peters following his FPAI visit, Evang showed some impatience for India’s population work, arguing that ‘the organisation and personnel for planned parenthood activities should be expanded as quickly as possible.’ India, however, was ‘on the right way,’ and Evang was ‘very much impressed indeed’ with the population work.^107^ Evang’s slight impatience points to an imbalance between Western policymakers’ ambitions for Indian population control, and Indian perceptions of the pace and methods by which this should proceed. Shortly after Evang’s visit, Gore wrote to Ottesen-Jensen that ‘the Western Nations though aware of some of our problems, they are not fully conscious of the difference in attitude and approach in this country,’ indicating that Indian approaches needed to differ from Western ones, whilst India, as ‘a poor and uneducated country we have greatly to rely on the generosity of the Western Countries.’^108^

Thus, FPAI members could experience tensions between a reliance on Western funding, and a struggle to gain acceptance for cultural differences on birth control. The exchanges with the FPAI influenced Evang’s views, as he wrote to Peters that Scandinavian positions on India’s population problem had been marked by misrepresentations, and he now worked to correct these. Further, he noted:

Even if the methods at present available are not ideal for Indian conditions,…the education which takes place through the planned parenthood organization, through the maternal and child welfare centres etc. contribute a great deal towards …the emancipation of the Indian women.^109^

Indeed, these views were echoed in Evang’s report to the WHO, where he lauded the Indian authorities and medical profession and, above all, the FPAI and their ‘pioneering’ and ‘excellent’ work. Evang, used to meeting resistance when raising birth control, argued that the ‘the irrational inhibitions so often found in western countries did not seem to exist’ in India, and ‘an open and frank interest was displayed.’^110^

## Evang’s and the Visiting Team’s work: possible outcomes

Evang worked to influence both Indian and WHO opinions. However, it is difficult to establish any direct, specific impact of Evang’s participation in the WHO’s Visiting Team on India’s health work. Firstly, Evang disseminated his views on social medicine and public health in India through numerous lectures and meetings, as well as media. On All India Radio, Evang talked on ‘Social Aspects of Medical Science,’^111^ and he offered the Norwegian broadcasting corporation NRK talks of his experiences in India.^112^ Also, A.P. Pillay requested a condensed version of Evang’s talk on ‘Health problems in relation to Human Reproduction’ for the *International Journal of Sexology*’s May 1953 issue,^113^ while Evang’s lecture ‘Role of the Medical Profession in Forming Nutrition Policy’ appeared in *The Madras Medical College Magazine’s W.H.O. Special Number.* ^114^ His dissemination efforts seem to have at least made an impression, as after his trip, Evang received news from the Director of Public Health in Poona, D.K. Viswanathan, that A.B. Shetty, the Minister for Health in Madras was now ‘full of enthusiasm for public health, a rather welcome change, and I believe that the etiologic agent in this instance is of Norwegian origin.’ Viswanathan added that other ministers were also ‘convinced on the need for greater emphasis being laid on preventive medicine.’^115^

Secondly, in late spring 1953 Evang submitted his report about public health in India to the WHO, where he gave his recommendations for India’s health work, and for which he received much praise.^116^ The report’s main concern was the need to strengthen preventive medicine vis-à-vis curative medicine. Evang had found a ‘present strong emphasis for curative medicine in India’, which meant that the position of those working in preventive medicine was ‘not too strong and they were working against heavy odds.’^117^ Thus, Evang’s top recommendation was a ‘Merger of the “Medical” and “Public Health” branches of the health administration into one unified administration of Health Services, headed by medical men with training in Public Health, social, preventive and administrative medicine and epidemiology.’^118^ This merger was to take place at all levels, from local to central. According to Evang, a ‘fundamental reorientation seems essential to switch the emphasis from curative to preventive medicine.’ To Evang’s mind, ‘establishment even of the best curative services in India…would not considerably (or perhaps not at all) improve health conditions of the country.’ Other areas for prioritisation were sanitation, sick insurance, drug production, maternal and child health, family planning and statistical work.^119^ Evang’s recommendations may have resulted from his reactions to what Niels Brimnes shows was a main tendency of India’s health work during the country’s first decade; mirroring international changes in health, India gradually prioritised vertical, technology-focused approaches over broader, horizontal ones.^120^

Evang’s report appealed to C. Mani, the WHO’s Regional Director for South East Asia, who was ‘glad to find in it your strong support for the focussing of attention on the primary health needs in the preventive field as against the present craze for more and more curative medicine.’^121^ Mani had pleaded with the WHO Regional Committee for a shift in emphasis from curative to preventive medicine through his 1952 and 1953 reports,^122^ and he shared many of Evang’s views. Whilst generally, horizontal approaches were on the decline and vertical approaches on the rise within international health, Packard argues that the early years of the WHO was still marked by the presence and participation of supporters of social medicine, such as Evang, and perhaps most prominently, Brock Chisholm.^123^ Thus Evang’s views on the need to strengthen preventive vis-à-vis curative medicine, may indeed have enjoyed some support among a few core staff at the WHO.

Thirdly, on his return to Norway, Evang reached out to several of his Indian counterparts and connections, sharing his views. Writing to nearly 30 individuals working in India’s health system in various capacities, from Indian Minister of Health in Delhi Sushila Nayar to local GPs, Evang wrote both formal thank-you notes but also longer letters with his opinions and recommendations, and some of these led to longer exchanges and sustained correspondence on topics of common interest.^124^ Writing to the deans of Madras Medical College and Topiwalla National Medical College, Dr Prasada Rao and Dr S.G. Vengsarkar, Evang underlined that it was a

special pleasure and encouragement for me to learn the progressive and modern views which you held on medical education. Especially I think it is of vital importance to India that the present over-emphasis on clinical and curative medicine is balanced as quickly as possible by greater emphasis on preventive, social and constructive medicine. The more I saw of your country, the stronger I was struck by this, and now…, it stands out even clearer.^125^

Evang suggested one ‘supply every medical college in India which has not already got it, with a chair of preventive and social medicine,’ and had a concrete solution: ‘a young American specialist in this field, Milton Roemer’, whose WHO contract was coming to an end, might be of help. Roemer would be ‘very well suited just for the type of teaching which several universities in India would need,’ and since the ‘WHO is interested in promoting these branches of medicine, I do think that a request from an Indian university to WHO asking for the services of Dr. Roemer (…) might meet with approval.’^126^

In 1954, S.G. Vengsarkar replied that both Central and State authorities for some time had considered the creation of Social and Preventive Medicine departments. A ‘Comprehensive Medical Care Plan’ would be implemented by Topiwala National Medical College’s Preventive and Social Medicine Department, as well as by other hospitals. Indeed, the Government of India was approaching the WHO with a request for ‘the services of an expert to head and organise the Department of Preventive and Social Medicine at these institutions,’ and Vengsarkar asked Evang ‘to kindly do the best you can in this matter.’^127^

Evang remained disappointed in Indian authorities’ decision to reduce the numbers of the Visiting Team’s public health group, writing to colleagues that, ‘It is thus a sad mismatch that we are only two public health people in a group of 13. It should have been the contrary, so that we could have been of much greater use.’^128^ At the same time, Evang was optimistic as the Team had been met with great enthusiasm, and he argued that he had ‘sown a few seeds.’^129^

From the mid 1950s, social and preventive medicine did indeed gain some attention in Indian medical education, with the assistance of the WHO. Confirming S.G. Vengsarkar’s report to Evang, from 1954–1955, the Government of India initiated a scheme to establish departments of social and preventive medicine in medical colleges, as part of the first Five-Year Plan (1951–1956). The scheme likely had support at the highest political levels in the Government; writing about Indian medical education in 1960, Rajkumari Amrit Kaur argued that ‘the department of Preventive and Social Medicine in the training of the medical student is one of paramount importance in India.’ In colleges and hospitals, there should be the ‘fullest possible collaboration between the Department of Preventive and Social Medicine and other departments.’^130^

For the Second Five-Year Plan (1956–1961), the Government of India allocated Rs 2.5 lakhs (250 000) to the scheme for social and preventive medicine.^131^ By 1958, eleven such departments had been established in Indian medical colleges, Madras Medical College being one of these.^132^ From 1956 onwards, the work was given WHO funding, which established chairs of preventive and social medicine at Indian medical colleges.^133^ A core aim was to integrate preventive and social medicine with all medical subjects, also in the general curriculum, to create preventive and social medicine courses and give Indian doctors training at Harvard School of Public Health.^134^ By 1958, five ‘medical men’ had been ‘sent under the W.H.O. Fellowship Scheme’ to Harvard.^135^ WHO also sponsored visiting fellowships for international professors to set up new departments of social and preventive medicine, raise awareness and knowledge, and train local counterparts. By 1958, WHO-appointed experts had organised the preventive and social medicine departments of the Assam Medical College, Dibrugarh, and the Medical College, Nagpur, Maharashtra.^136^ In 1956, Axel Höjer was offered a post as WHO-financed visiting professor at Assam Medical College by Rajkumari Amrit Kaur, who was impressed with his social medical work.^137^ At the same time, WHO public health fellowships were increasing rapidly in the 1950s,^138^ and thus Indian use of these may not be attributed to Evang’s work in India.

The India–WHO collaboration on India’s scheme for social and preventive medicine from the mid-1950s onwards may, however, point to what Packard has shown was a feature of the WHO’s early years; that ‘the broader vision of health held by WHO’s founding architects was also represented in the early activities of this organization.’^139^ According to Packard, social medicine’s continued influence is shown by the key roles played by proponents of the approach on WHO expert committees, such as Karl Evang’s chairing of the Expert Committee on Public Health Administration.^140^ While these influences rapidly waned through the 1950s, such observations nevertheless complicate the narrative of early post-war international health work, pointing towards a coexistence, albeit short-lived, of both horizontal and vertical perspectives, of social and technological approaches in health.

Finally, one outcome of Evang’s Visiting Team participation, and his numerous meetings, lectures, field visits and exchanges, was that it left one person forever changed: Evang himself. A striking, recurring feature of Evang’s archives is his vivid description of the multitudes of impressions from India. In his frequent correspondence, Evang expressed strong admiration for the Indian medical services and practitioners. At the same time, however, Evang was deeply concerned about what he characterised as ‘a combination of abysmal ignorance and incompetence on the one hand, and excellent solutions and outstanding efforts on the other.’^141^ Puzzled by what he saw as a rupture between preventive and curative medicine, Evang argued that those in clinical medicine ‘concern themselves with a few, preferably odd and rare cases, discussing the finer points of treatment…whilst epidemics are raging, right outside their doors.’^142^ Reporting to colleagues at home, Evang argued that ‘it is absolutely clear that this is worth the stay,’^143^ and that India was ‘absolutely fantastic’^144^ and ‘a key country in so many respects…necessary to see in order to understand the world today.’^145^

## Conclusions

This article has examined early postcolonial international health work, through the writings of Karl Evang and the 1953 WHO Visiting Team of Medical Scientists to India. What can the Team’s work in India tell us about WHO health cooperation in the early 1950s? To Evang, the participation in the WHO Visiting Team entailed comprehensive interaction with Indian counterparts in medicine, administration and politics, at central, state and local levels, and provided numerous opportunities for exchanges of views, in line with the WHO’s intentions. Some of these interactions were followed up after the Team’s work had finished, through continued correspondence and efforts to cooperate. Evang’s own descriptions certainly paint a picture of a visiting medical scientist who learnt vast amounts from his Indian counterparts. At the same time, Evang never hesitated to defend his views, when these clashed with those of his Indian counterparts. When Indian authorities refused the WHO’s original team composition, this had several WHO representatives up in arms. Thus, while the change in name from ‘Medical Teaching Mission’ to ‘Visiting Team of Medical Scientists’ signalled a WHO intention to move away from a missionary approach towards dialogue and exchange, Evang’s and the Visiting Team’s examples show us that while exchanges and dialogues indeed were extensive, these nevertheless hardly took place on a genuinely equal footing. Thus, the WHO and its Visiting Teams seemingly still had some way to go before the aim of ‘equal exchange of views’ between visitors and hosts was entirely achieved.

From Karl Evang’s vantage point, the article has also explored the role and legacy of social medicine in early post-war international health work. Evang’s WHO Field Diary, his programme in India, and his correspondence before, during and after the trip, all indicate that Evang consistently kept an eye on social and preventive medicine. Also, Evang’s view of an imbalance between clinical and curative, and social and preventive health in India, and the need to address this, seemingly had support of some core staff in the WHO, evidenced by Mani’s wholehearted approval of Evang’s report. Furthermore, such perceptions seem reflected in the WHO’s Information Bulletins, which emphasised that ‘the preventive approach of the public health specialists has to find its legitimate place in any and every branch of the basic and clinical sciences.’^146^ Several recent contributions have pointed to the ‘narrowing vision’ of the WHO during the 1950s, and indeed also the Indian mirror image of this vision, which entailed a move towards vertical intervention in international health. Evang’s experiences in the WHO Visiting Team, however, offer a slightly diverging impression; the ideas and opinions of an actor committed to prioritising and promoting social and preventive medicine, and who seemingly expected the WHO to support his views. Indeed, the Government of India’s 1954–1955 emphasis on social and preventive medicine in medical education may seem to run counter to the idea of India’s policies of health and development reflecting international ideational developments and turning towards technology-focused approaches. It should be borne in mind, however, that India’s modernizing efforts comprised a broad range of approaches, and that ideas of development had considerable flexibility, accommodating compromises.^147^ Thus, the ongoing overall turn towards technology-focused vertical interventions did not necessarily preclude some instances of more horizontally focused approaches existing side-by side, in parallel.

Using Evang’s experiences from the 1953 WHO Team’s work in India as our lens on early post-war international health thus complicates dominant narratives and may further nuance understandings of the role and legacy of social medicine in the WHO during a period of reorientation in international health. Indeed, while social medicine’s influence waned within the WHO, actors such as Karl Evang remained in key health positions until the 1970s – the decade when horizontal approaches returned to international prominence through the focus on primary health care – and Evang applauded their return.^148^

Finally, through examples such as Evang’s we may make global health histories more diverse, including the perspectives of actors hailing from smaller but still central players in international health. A Scandinavian internationalist with a radical political background, Karl Evang’s enduring participation in international health, across the Second World War, also challenges traditional chronologies, showing the continuity of actors and approaches in medical work, beyond changing organisational landscapes and established historiographical periodisations. Thus, inclusion of a plurality of perspectives may offset, decentre and challenge, but also fruitfully nuance and complement dominant narratives of development, health and international relations.

## Footnotes

1 Norway’s national archives (*Riksarkivet*) (NO RA) personal archives (*personarkiv*) (PA) 0386 Series M, 152, Medical Scientists India (MSI): Editorial by A. Varadarajasundaram, *The Madras Medical College Magazine, WHO Special Number*, 3.

2 NO RA PA 0386 Series M, 152, MSI: WHO Visiting Team of Medical Scientists, Information Bulletin No. 1, 26 May 1952.

3 Several histories of international health mention Evang as a central actor in the WHO’s early years. See Randall M. Packard , A History of Global Health. Interventions into the Lives of Other Peoples (Baltimore: Johns Hopkins University Press, 2016) and Marcos Cueto , Theodore M. Brown and Elizabeth Fee , The World Health Organization. A History (Cambridge: Cambridge University Press, 2019).

4 See Karl Evang , Verdens helseorganisasjon (Bergen: John Griegs Forlag, 1949), 84–8.

5 See eg. Tore Grønlie , Sentraladministrasjonens historie etter 1945. Ekspansjonsbyråkratiets tid 1945–1980 (Bergen: Fagbokforlaget, 2009); Øivind Larsen , ‘Karl Evang – det opplyste eneveldets siste representant?’, Michael, 13, 3 (2016), 221–6; Trond Nordby , Karl Evang. En biografi (Oslo: Aschehoug, 1989); Rune Slagstad , De nasjonale strateger (Oslo: Pax, 1998) and Siv Frøydis Berg , Den unge Karl Evang og utvidelsen av helsebegrepet (Oslo: Solum forlag, 2002).

6 Examples from vast fields of research include James C. Scott , Seeing Like a State. How Certain Schemes to Improve the Human Condition have Failed (New Haven: Yale University Press, 1999); Matthew Connelly , ‘Seeing Beyond the State: The Population Control Movement and the Problem of Sovereignty’, Past & Present, 193, 1 (2006), 197–233 ; Anne-Emanuelle Birn, ‘WHOse health agenda? 70 years of struggle over WHO’s mandate’, *The Lancet*, 391, 10128 (2018), 1350–1; Cueto, Brown and Fee, *op. cit.* (note 4); Packard, *op. cit.* (note 4).

7 See eg. Sunil Amrith , Decolonizing International Health. India and Southeast Asia, 1930–65 (London: Palgrave, 2006); A. Raza , F. Roy and B. Zachariah (eds), The Internationalist Moment: South Asia, Worlds, and World Views, 1917–39 (New Delhi: Sage, 2015); David C. Engerman , The Price of Aid. The Economic Cold War in India (Cambridge: Harvard University Press, 2018).

8 Amrith, *op. cit.* (note 8), 14–5; Sunil Amrith ‘Internationalising health in the twentieth century’ in G. Sluga and P. Clavin (eds), Internationalisms. A Twentieth-Century History (Cambridge: Cambridge University Press, 2017), 256–7.

9

Sanjoy Bhattacharya , ‘The World Health Organization and Global Smallpox Eradication’, Journal of Epidemiology and Community Health, 62, 10 (2008), 909–121879104910.1136/jech.2006.055590PMC2602749; Sanjoy Bhattacharya , ‘International Health and the Limits of its Global Influence: Bhutan and the Worldwide Smallpox Eradication Programme’, Medical History, 57, 4 (2013), 461–862406991310.1017/mdh.2013.63PMC3865968.

10 Discussion has revolved around liberalism in India, but it has gradually also included the translation of political ideas into political practice. See eg. Uday Singh Mehta , Liberalism and Empire: A Study in Nineteenth-Century British Liberal Thought (Chicago: University of Chicago Press, 1999); Benjamin Zachariah , Developing India: An Intellectual and Social History, c. 1930–50 (New Delhi: Oxford University Press, 2005); Christopher A. Baily , Recovering Liberties: Indian Thought in the Age of Liberalism and Empire (Cambridge: Cambridge University Press, 2012); Partha Chatterjee , The Black Hole of Empire: History of a Global Practice of Power (Princeton: Princeton University Press, 2012); Neilesh Bose , ‘The Cannibalized Career of Liberalism in India’, Modern Intellectual History, 12, 2 (2015), 475–84; Christopher A. Baily , ‘The Ends of Liberalism and the Political Thought of Nehru’s India’, Modern Intellectual History, 12, 3 (2015), 505–26.

11 See S. Kapila , ‘Global intellectual history and the Indian political’, in D. McMahon and S. Moyn (eds), Rethinking Modern European Intellectual History (New York: Oxford University Press, 2014), 253–74; Shruti Kapila , Violent Fraternity. Indian Political Thought in the Global Age (Princeton: Princeton University Press, 2021).

12 I am indebted to *Medical History*’s reviewers for thoughtful insights and even turns of phrase expressed in this paragraph.

13

Iris Borowy , Coming to Terms with World Health. The League of Nations Health Organisation 1921-1946 (Frankfurt am Main: Peter Lang, 2009), 21–2.

14 Sunil Amrith, *op. cit.* (note 8), 47–56, 93, 189–90. See also Niels Brimnes , Languished Hopes. Tuberculosis, the State and International Assistance in Twentieth-century India (New Delhi: Orient Black Swan, 2016), 86–7.

15 Cueto, Brown and Fee, *op. cit.* (note 4), 62–4.

16 Sanjoy Bhattacharya, ‘Introduction’, *Economic and Political Weekly*, 43, 12/13 (2008), 39–40. See also Sunil Amrith, *op. cit.* (note 9), 245–64.

17

Helge Pharo , Hjelp til selvhjelp: det indisk-norske fiskeriprosjektets historie 1952–72 (Oslo: Norsk utenrikspolitisk institutt, 1986).

18

Patricia Clavin , ‘Time, Manner, Place: Writing Modern European History in Global, Transnational and International Contexts’, European History Quarterly, 40, 4 (2010), 624–40.

19 Bhattacharya, ‘International Health’ *op. cit.* (note 10).

20 Established in 1921, *Mot Dag* was affiliated with the Communist Party 1927–29 and disbanded in 1935.

21

Karl Evang , Fødselsregulering. Dens berettigelse og dens praktiske gjennemførelse (Oslo: Fram forlag, 1930).

22 Nordby, *op. cit.* (note 6), 47.

23
*Ibid.*, 57; Evang, *Verdens, op. cit.* (note 5).

24

Aina Schiøtz , ‘Mellom legekunst og vitenskap – Tidsskriftet 1906–56’, Tidsskrift for Den norske legeforening, 126, 24 (2006), 3300–417170793.

25

Per Fugelli et al., ‘Sosialmedisin – på sporet av det tapte fag’, Tidsskrift for Den norske legeforening, 120, 25 (2000), 3057–6111109398; Øivind Larsen (ed.), Norges Leger. Bind IV (Oslo: Den norske lægeforening, 1996), 669–70.

26

Øivind Larsen (ed.), Norges Leger. Bind IV (Oslo: Den norske lægeforening, 1996), 669–70.

27 Berg, *op. cit.* (note 6), 35.

28
*Ibid.*, 37; Swedish *Socialistiska Medicinärförening* and Danish *Socialistisk Medicinergruppe.*

29 Fugelli, *op. cit*, (note 26), 3057–61; Magne Nylenna , ‘Hva er samfunnsmedisin?’, Michael, 13, 2 (2016), 79–85 ; Berg, *op. cit.* (note 6), 34.

30 Berg, *op. cit.* (note 6), 19.

31

Karl Evang , ‘Lægestanden og socialismen’, Meddelsesblad for Socialistiske Lægers Forening, 1, (1932), 6–11 , quoted in Berg, *op. cit.* (note 6), 43.

32 See Kari Hernæs Nordberg, ‘Om nytelse og nytte. Seksualitet i “Populært Tidsskrift for Seksuell Oplysning”’ (unpublished MA thesis: University of Oslo, 2003).

33 See Lena Lennerhed , Sex i folkhemmet. RFSUs tidiga historia (Uppsala: Gidlunds förlag, 2002); Linder, Doris , Crusader for Sex Education. Elise Ottesen-Jensen (1886–1973) in Scandinavia and on the International Scene (Lanham: University Press of America, 1996).

34

Karl Evang and Otto Galtung Hansen , Norsk kosthold i små hjem. Virkelighet og fremtidsmål (Oslo: Tiden norsk forlag, 1937).

35 League of Nations, ‘Survey of National Nutrition Policies, 1937/38’, (Geneva, 1938), 11, available at https://biblio-archive.unog.ch/Dateien/CouncilMSD/C-478-M-321-1938-II-A_EN.pdf. (accessed 24.08.21); see also NO RA PA 0386 Series M, 152, MSI: Evang’s CV, 3.

36 See eg. Paul Weindling (ed.), International Health Organisations and Movements, 1918–1939 (Cambridge: Cambridge University Press, 1995).

37
*Ibid.*; Iris Borowy, *op. cit.* (note 14), 354–6; Josep L. Barona , The Rockefeller Foundation, Public Health and International Diplomacy, 1920–194 (London: Pickering & Chatto, 2015); Theodore Brown and Elizabeth Fee , ‘The Bandoeng Conference in 1937: A Milestone in Health, American Journal of Public Health, 98, 1 (2008), 42–31804877610.2105/AJPH.2007.119222PMC2156053.

38

Rune Slagstad , ‘De politiserende embetsmenn. En tradisjon i norsk forvaltning’, Norsk Administrativt Tidsskrift, 88, 3 (2011), 177 .

39 Fugelli, *op. cit.* (note 26), 3057–61; Nylenna, *op. cit.* (note 30), 79–85.

40 Larsen, *op. cit.* (note 27), 244.

41 Nordby, *op.cit,* (note 6), 19.

42 See Sunniva Engh, ‘Rockefeller Foundation og etableringen av Statens institutt for folkehelsen. Idéer, investeringer og institusjonsbygging i internasjonal helse, 1923–1935’, *Michael*, 16, 1 (2019), 10–35. Evang received Rockefeller Foundation funding in 1945 and 1947. See Rockefeller Archive Center (RAC), Rockefeller Foundation (RF), Record Group (RG) 1.1, Series 767K Norway, Sub-series State Inst. of Public Health, Oslo, 1945–1949, 1951, Box 3, Folder 31: The RF Health Commission resolution HC 45022 dated 14 December 1945, and The RF Health Commission resolution HC 45022 dated 28 March 1947 and 12 December 1947.

43

Karl Evang , ‘The United Nations Conference on Food and Agriculture, Hot Springs, Virginia, 18^th^ May-3^rd^ June 1943’, in D.M. Lyon, ‘The Hot Springs Conference’, Proceedings of the Nutrition Society, 2, 3-4 (1944), 163–6.

44

Karl Evang , ‘Today’s Global Frontiers in Public Health: Discussion’, American Journal of Public Health, 35, 2 (1945), 114–610.2105/ajph.35.2.89PMC162538018016122.

45 NO RA PA 0386 Series M, 152, MSI: Evang’s CV, 3.

46 Cueto, Brown and Fee focus on Sze and de Souza’s roles behind the idea of the WHO, omitting Evang’s participation. Cueto, Brown and Fee, *op. cit.* (note 4), 37–8. Similarly, see Amrith, *op. cit.* (note 8), 75–6, and Amy L.S. Staples, *The Birth of Development. How the World Bank, Food and Agriculture Organization, and World Health Organization Changed the World, 1945–1965* (Kent: Kent State University Press, 2006), 132. The idea of an international health organisation, however, originated in a meeting between Evang, de Souza and Sze 2 May 1945. Szeming Sze’s diary, 1945, 1, 9, 16, available online: https://digital.library.pitt.edu/islandora/object/pitt%3A31735066261466/ (accessed 24.08.2021). See also Szeming Sze, *The Origins of the World Health Organization. A Personal Memoir, 1945–1948* (Florida: L.I.S.Z. Publications, 1982), 2, 5.

47 NO RA PA 0386 Series M, 152, MSI: Evang’s CV, 3.

48 NO RA PA 0386 Series M, 153, MSI: Evang to Mathiesen, 10 March 1953.

49 Sze, *The Origins of the World Health Organization. op. cit.* (note 28), 20.

50 Evang was a member of the FAO’s Standing Committee on Nutrition 1945–47. NO RA PA 0386 Series M, 152, MSI: Evang’s CV, 3.

51 See S. Engh , ‘Georg Borgström and the population-food dilemma. Reception and consequences in Norwegian public debate in the 1950s and 1960s’, in J. Östling , N. Olsen and D. L. Heidenblad (eds), Histories of Knowledge in Postwar Scandinavia. Actors, Arenas, and Aspirations (London: Routledge, 2020), 39–58 .

52 See Matthew Connelly , Fatal Misconception: The Struggle to Control World Population (Harvard: Belknap Press, 2008).

53 NO RA PA 0386 Series M, 143, WHO – Helseforsamlinger 1952: WHO Fifth World Health Assembly, Committee on Programme and Budget, Provisional Minutes of the Eighth Meeting, 13 May 1952, 4. See also Packard, *op. cit.* (note 4), 197–8.

54
*Ibid.*; see also John Farley , Brock Chisholm, the World Health Organization, and the Cold War (Vancouver: UBC Press, 2008), 171–84.

55 See Executive Board, 1 and 2 (1948), *Reports of the Executive Board: First and Second Sessions Held in Geneva from 16 to 28 July and from 25 October to 11 November 1948*, available at https://apps.who.int/iris/handle/10665/85593; Executive Board, 3 (1949), *Report of the Executive Board: Third Session Held in Geneva from 21 February to 9 March 1949*, available at https://apps.who.int/iris/handle/10665/85596; Executive Board, 4 (1949), *Report of the Executive Board: Fourth Session Held in Geneva from 8 to 19 July 1949: Supplement: Reports of the Joint Committee on Health Policy, UNICEF/WHO*, available at https://apps.who.int/iris/handle/10665/85601; Executive Board, 5 (1950). *Report of the Executive Board: Fifth Session Held in Geneva from 16 January to 2 February 1950: Part I*, available at https://apps.who.int/iris/handle/10665/85604 (All links accessed 05.10. 2021).

56 Annika Berg, *Den gränslösa hälsan. Signe och Axel Höjer, folkhälsan och expertisen* (PhD Thesis: Uppsala University, 2009), 350–5.

57

Karen Gram-Skjoldager , Haakon Andreas Ikonomou and Torsten Kahlert , ‘Scandinavians and the League of Nations Secretariat, 1919–1946’, Scandinavian Journal of History, 44, 4 (2019), 454–83.

58 NO RA PA 0386 Series M, 152, MSI: Kohn to Evang, 28 March 1952.

59 NO RA PA 0386 Series M, 152, MSI: Evang to Kohn, 2 April 1952.

60 Ibid.

61 NO RA PA 0386 Series M, 152, MSI: Kohn to Evang, 8 April 1952.

62 NO RA PA 0386 Series M, 152, MSI: WHO Visiting Team of Medical Scientists, Information Bulletin No. 1, 26 May 1952.

63
*Ibid.*, 2.

64
*Ibid.*

65
*Ibid.*

66 The final team included Professor Einar Lundesgaard (University of Copenhagen), Professor Corneille Heymans (University of Ghent), Sir Alexander Fleming (The Wright-Fleming Institute), Professor Joseph C. Aub (Harvard University), Professor Samuel Z. Levine (Cornell University), Professor Pietro Valdoni (University of Rome), Professor Erik Husfeldt (University of Copenhagen), Dr Henry Osmond-Clarke (University of London), Professor Georges Portmann (University of Bordeaux), Professor Stuart C. Cullen (The State University of Iowa), Professor Leo G. Riegler, (University of Minnesota), Professor Edward Grzegorzewski (Director of WHO Division of Education and Training Services), Dr Karl Evang (Director General of Public Health, Norway), Professor John E. Gordon (Harvard School of Public Health, Dorothy E. Snavely (Director, USC), Frank J. Deveau (WHO), and Shirley Fuller Brooks (Secretary). NO RA PA 0386 Series M, 152, MSI: Visiting Team of Medical Scientists, Madras Programme.

67 NO RA PA 0386 Series M, 152, MSI: WHO Visiting Team of Medical Scientists, Information Bulletin No. 2, 2 June 1952.

68
*Ibid.*

69
*Ibid.*

70 NO RA PA 0386 Series M, 152, MSI: See WHO Visiting Team of Medical Scientists, Information Bulletins No. 3–10.

71 NO RA PA 0386 Series M, 152, MSI: Eliassen to Kohn 18 August 1952, and Eliassen to Evang, 21 August 1952.

72 NO RA PA 0386 Series M, 152, MSI: Evang to Kohn, 11 September 1952.

73 NO RA PA 0386 Series M, 152, MSI: Evang to Gordon, 17 October 1952, and Gordon to Evang, 6 November 1952.

74 NO RA PA 0386 Series M, 152, MSI: Kohn to Evang, 17 September 1952.

75 NO RA PA 0386 Series M, 152, MSI: Evang to Kohn, 17 October 1952.

76 NO RA PA 0386 Series M, 152, MSI: Kohn to Evang, 21 October 1952, and Evang to Gordon, 29 December 1952.

77 Bhore Committee, *Report of the Health Survey and Development Committee*, Vol. II (Calcutta: Government of India Press, 1946), 6–17.

78 Amrith, *op. cit.* (note 8), 56–63.

79
*Ibid.*; Brimnes, *op. cit.* (note 15), 63; see also Roger Jeffrey , The Politics of Health in India (Berkeley: University of California Press, 1988).

80

Sunil Amrith , ‘Political Culture of Health in India. A Historical Perspective’, Economic and Political Weekly, 42, 2 (2007), 114–20.

81

Karl Evang , ‘Noen aktuelle oppgaver ved gjenreisningen av den norske folkehelsen og det norske helsevesen’, Tidsskrift for Den norske lægeforening, 65 (1945), 266–71.

82 See Nordby, *op. cit.* (note 6), 119; Trond Nordby , ‘Helsedirektør Evangs planer for velferdsstaten’, Michael, 6 (2009), 331–7; A. Berg and T. Ryymin , ‘The people’s health, the nation’s health, the world’s health. *Folkhälsa* and *folkehelse* in the writings of Axel Höjer and Karl Evang’ in J. Kananen , S. Bergenheim and M. Wessel (eds), Conceptualising Public Health. Historical and Contemporary Struggles over Key Concepts (Abingdon: Routledge, 2018), 76–100 .

83 Evang, *op. cit.* (note 45), 115.

84 Berg, *op. cit.* (note 57), 364–5.

85

Nikhil Menon , Planning Democracy. Modern India’s Quest for Development (Cambridge, Cambridge University Press, 2022), 17 ; see also Zachariah, *op. cit.* (note 11); Sunil Khilnani , The Idea of India (London: Penguin Books, 2012); Ramachandra Guha , India after Gandhi. The History of the World’s Largest Democracy (London: Pan Books, 2008); Manu Goswami , Producing India: from Colonial Economy to National Space (Chicago: University of Chicago Press, 2004).

86

Corinna Unger , ‘Towards Equilibrium: American Foundations and Indian Modernization, 1950s to 1970s’, Journal of Global History, 6, 1 (2011), 129 .

87 Zachariah, *op. cit.* (note 11).

88 Unger, *op. cit.* (note 87), 132.

89 See Sanjoy Bhattacharya , Expunging Variola: The Control and Eradication of Smallpox in India, 1947–1977 (New Delhi: Orient Longman, 2006), Ch. 1.

90 NO RA PA 0386 Series M, 152, MSI: Visiting Team of Medical Scientists, Bombay Programme, and Schedule of Visiting Team of Medical Scientists to India (Madras Phase).

91 NO RA PA 0386 Series M, 152, MSI: Evang’s WHO Field Diary 17–22 March 1953.

92 Bhore Committee, *Report of the Health Survey and Development Committee,* Vol. I-III (Calcutta: Government of India Press, 1946).

93 NO RA PA 0386 Series M, 152, MSI: Raja to Evang, 18 March 1953, and Appendix II: A note on the Teaching of Preventive Medicine by K.C.K.E. Raja. From 1956–59, Raja was the Director of the International Institute for Population Studies in Bombay, and he was also a member of the 1961 Mudaliar Committee, see *Report of the Health Survey and Planning Committee (August 1959–October 1961)* (Government of India, 1962).

94 NO RA PA 0386 Series M, 152, MSI: Evang’s WHO Field Diary 9–16 and 23–29 March 1953.

95 NO RA PA 0386 Series M, 152, MSI: Diary of Evang, 10 March and 23 March 1953.

96 NO RA PA 0386 Series M, 153, MSI: Evang to Bjørnsson, 10 March 1953.

97 NO RA PA 0386 Series M, 152, MSI: Signe and Axel Höjer to Evang, 18 August 1952.

98
*Ibid.*

99 NO RA PA 0386 Series M, 152, MSI: Evang to Signe and Axel Höjer, 2 September 1952.

100 See eg. Dhanvanthi Rama Rau , An Inheritance: The Memoirs of Dhanvanthi Rama Rau (London: Harper and Row, 1977); Avabai Wadia , The Light is Ours: Memoirs and Movements (London: IPPF, 2001); Sanjam Ahluwalia , Reproductive Restraints. Birth Control in India, 1877–1947 (Urbana: University of Illinois Press, 2008).

101 NO RA PA 0386 Series M, 152, MSI: Diary of Evang, 10 March 1953.

102 NO RA PA 0386 Series M, 153, MSI: Rama Rau to Evang, 13 March 1953; NO RA PA 0386 Series M, vol 152, MSI: Evang’s WHO Field Diary for 23 March 1953.

103 NO RA PA 0386 Series M, 152, MSI: Visiting Team of Medical Scientists Programme, Bombay.

104 Rama Rau, *op.cit.* (note 100), 253–67.

105 Maren Bakken, ‘«Ut! Ut och beretta det!» Elise Ottesen-Jensen’s internasjonale engasjement’ (unpublished MA thesis: University of Oslo, 2018). See Wadia, *op. cit.* (note 100), 228.

106 Wadia, *op. cit.* (note 100), 240.

107 NO RA PA 0386 Series M, 152, MSI: Evang to Peters, 18 April 1953.

108 Gore to Ottesen-Jensen, 16 May 1953, as quoted in Bakken, *op. cit.* (note 105), 63-4.

109 NO RA PA 0386 Series M, 152, MSI: Evang to Peters, 18 April 1953.

110 NO RA PA 0386 Series M, 153, MSI: Evang to Chisholm, 14 April 1953, 67–69.

111 NO RA PA 0386 Series M, 153, MSI: All India Radio to Evang, 16 March 1953.

112 NO RA PA 0386 Series M, 153, MSI: Fostervoll to Evang, 12 March 1953.

113 NO RA PA 0386 Series M, 153, MSI: Pillay to Evang, 13 March 1953.

114 NO RA PA 0386 Series M, 152, MSI: Karl Evang, ‘Role of the Medical Profession in Forming Nutrition Policy’, *The Madras Medical College Magazine, W.H.O. Special Number*, I–XVI.

115 NO RA PA 0386 Series M, 152, MSI: Viswanathan to Evang, 30 April 1953.

116 NO RA PA 0386 Series M, 152, MSI: Vengsarkar to Evang, 24 August 1954.

117 NO RA PA 0386 Series M, 153, MSI: Evang to Chisholm, 14 April 1953, 6. Similar views expressed on pages 13, 14, 15, 22, 65–6.

118 NO RA PA 0386 Series M, 153, MSI: Evang to Chisholm, 14 April 1953, 69–70.

119
*Ibid.*

120 See Niels Brimnes , ‘Rallying around the magic wand: Visions of social medicine, public health and disease control in India 1946–1957’, in I. Borowy and B. Harris (eds), Yearbook for the History of Global Development (Boston: De Gruyter, 2023), 175–200 . For a critique of vertical approaches in India’s health service, see eg. Debabar Banerji , ‘The People and Health Service Development in India: A Brief Overview’, International Journal of Health Services, 34, 1 (2004), 123–421508867710.2190/9N5U-4NFK-FQDH-J46W.

121 NO RA PA 0386 Series M, 152, MSI: Mani to Evang, 27 July 1953.

122 NO RA PA 0386 Series M, 152, MSI: Regional Director’s Report, July 1951–July 1952 and Regional Director’s Report, July 1952–July 1953, C. Mani.

123 Packard, *op. cit.* (note 4), 99–102.

124 NO RA PA 0386 Series M, 152, MSI contains Evang’s correspondence.

125 NO RA PA 0386 Series M, 152, MSI: Evang to Rao, 24 April 1953 and Evang to Vengsarkar, 24 April 1953.

126
*Ibid.* In late 1952, the US government withdrew its support of Roemer’s work due to the influence of McCarthyism. See Emily K. Abel , Elizabeth Fee and Theodore M. Brown , ‘Milton I. Roemer Advocate of Social Medicine, International Health, and National Health Insurance’, American Journal of Public Health, 98, 9 (2008), 1596–71863307410.2105/AJPH.2008.134189PMC2509620.

127 NO RA PA 0386 Series M, 152, MSI: Vengsarkar to Evang, 24 August 1954.

128 NO RA PA 0386 Series M, 153, MSI: Evang to Ustvedt, 28 February 1953.

129 NO RA PA 0386 Series M, 153, MSI: Evang to Mellbye, 10 March 1953.

130 Rajkumari Amrit Kaur, ‘Medical Education in India’, *Postgraduate Medical Journal*, 36, 420 (1960), 592–7.

131 NO RA PA 0386 Series M, 142, WHO – helseforsamlinger 1957–1963: Swasht Hind, Ten Years of Health Progress, Vol II, No 3, March 1958, 76.

132
*Ibid.*

133 Berg, *op. cit.* (note 57), 389.

134
*Ibid.*

135 NO RA PA 0386 Series M, 142, WHO – helseforsamlinger, *op. cit.* (note132), 76.

136
*Ibid.* Assam Medical College still acknowledges the role of ‘WHO visiting Professor Dr. Alex J. Hogjer’ [sic.], see https://amch.edu.in/view/department/community+medicine (accessed 23.08.21).

137 Berg, *op. cit.* (note 57), 389.

138

Y. Lin , T. David and D. Rodogno , ‘Fellowship Programs for Public Health Development. The Rockefeller Foundation, UNRRA and the WHO (1920s–1970s)’, in L. Tournès and G. Scott-Smith (eds), Global Exchanges. Scholarships and Transnational Circulations in the Modern World (New York: Berghahn Books, 2018),140–55.

139 Packard, *op. cit.* (note 4), 101.

140 Packard, *op. cit.* (note 4), 101-4.

141 NO RA PA 0386 Series M, 153, MSI: Evang to Mellbye, 10 March 1953.

142 NO RA PA 0386 Series M, 153, MSI: Evang to Ustvedt, 28 February 1953.

143 NO RA PA 0386 Series M, 153, MSI: Evang to Bjørnsson, 10 March 1953.

144 NO RA PA 0386 Series M, 153, MSI: Evang to Mellbye, 10 March 1953.

145 NO RA PA 0386 Series M, 153, MSI: Evang to Eker, 10 March 1953. See also *Reisebrev* [Travel letters] 1–3, sent to Evang’s colleagues at the Directorate of Health, 10 March 1953, 26 February 1953 and 16 March 1953.

146 NO RA PA 0386 Series M, 152, MSI: WHO Visiting Team of Medical Scientists, Information Bulletin No. 1, 26 May 1952.

147 Unger, *op. cit.* (note 87); Zachariah, *op. cit.* (note 11); Brimnes, *op. cit.* (note 121).

148 Ebba Wergeland, ‘Det sosialmedisinske perspektivet – arven etter Karl Evang’, available at https://www.helsetilsynet.no/globalassets/opplastinger/om_helsetilsynet/karlevang/prisvinners_takketale_wergeland2012.pdf (accessed 24.08.21).

